# Development of a Rainbow Trout (*Oncorhynchus mykiss*) Intestinal *In Vitro* Platform for Profiling Amino Acid Digestion and Absorption of a Complete Diet

**DOI:** 10.3390/ani13142278

**Published:** 2023-07-12

**Authors:** Rolando Pasquariello, Radmila Pavlovic, Marcelo A. Chacon, Federica Camin, Nicole Verdile, Guro Løkka, Sara Panseri, Massimo Faustini, Amos Tandler, David Peggs, Trond M. Kortner, Amir Bitan, Tiziana A. L. Brevini, Fulvio Gandolfi

**Affiliations:** 1 Department of Agricultural and Environmental Sciences, University of Milan, 20133 Milan, Italy; rolando.pasquariello@unimi.it (R.P.); federica.camin@unimi.it (F.C.); nicole.verdile@unimi.it (N.V.); 2 Department of Veterinary Medicine and Animal Sciences, University of Milan, 26900 Lodi, Italy; radmila.pavlovic1@unimi.it (R.P.); sara.panseri@unimi.it (S.P.); massimo.faustini@unimi.it (M.F.); tiziana.brevini@unimi.it (T.A.L.B.); 3 Proteomics and Metabolomics Facility (ProMeFa), IRCCS San Raffaele Scientific Institute, 20132 Milan, Italy; 4 Israel Oceanographic and Limnological Research (IOLR), The National Center for Mariculture, Eilat 8800001, Israel; marcelo.chacon@ocean.org.il (M.A.C.); tandler@ocean.org.il (A.T.); amir.bitan@ocean.org.il (A.B.); 5 Department of Paraclinical Sciences, Faculty of Veterinary Medicine, Norwegian University of Life Sciences (NMBU), 1433 Ås, Norway; guro.lokka@nmbu.no (G.L.); trond.kortner@nmbu.no (T.M.K.); 6 Skretting Aquaculture Research Centre, 4016 Stavanger, Norway; david.peggs@skretting.com

**Keywords:** *in vitro* digestion, intestine, digestibility, bioavailability, aquaculture, sustainable feed

## Abstract

**Simple Summary:**

The generation of an artificial intestine could minimize the use of *in vivo* trials, which are presently necessary for testing aquafeeds. As well as being lengthy and expensive, they also require the use of many animals. Furthermore, although *in vivo* screening allows for a precise evaluation of the final effect of each feed, it does not improve our basic knowledge of the cellular and molecular mechanisms that determine the end results. This, in turn, severely limits our ability to understand and predict the biological value of ingredients, both individually and in differing combinations thereof. To circumvent these constraints, we previously derived and characterized two novel cell lines from the proximal (RTpi-MI) and distal intestine (RTdi-MI) of rainbow trout (*Oncorhynchus mykiss*), demonstrating that the two cell lines retain the main properties of the intestinal tracts from which they originate. In the present work, we used these cells to set up a functional intestinal barrier on a permeable membrane support to investigate the digestion and absorption of dietary amino acids in fish feed. The results demonstrate that the procedure we developed enables the quantification of individual amino acids that cross the intestinal wall. This new platform will now be used to screen and select new feed ingredients/formulations.

**Abstract:**

The ever-increasing number and variation of raw materials utilized to provide alternative feed formulations continues to allow for a more sustainable and flexible approach. Testing all these options *in vivo* is still the most robust and reliable manner to pick the best raw material candidates, but it requires the use of large numbers of animals and is time-consuming and expensive. Therefore, we are developing an *in vitro* platform that can provide a reliable evaluation of new ingredients. The main aim of this work was to combine an *in vitro* digestion protocol of extruded, commercially relevant aquafeeds with the exposure of intestinal epithelial cells to the extracted bio-available fraction (BAF). The results show that 250,000 cells/cm^2^ represents the optimal seeding density and that up to 50% BAF concentration for up to 24 h had no negative effects on the epithelial barrier morphology and function. It is possible to determine amino acid digestibility and bioavailability in all the experimental conditions (with and without BSA, at 25% and 50% dilution) and at all time points (0, 6, and 24 h). However, BAF concentration, the medium used for its dilution, and the length of exposure to the different epithelial cell lines can all influence the results and, therefore, must be selected according to the final aim of the experiment.

## 1. Introduction

To improve animal welfare, sustainability, and product quality in aquaculture, it is necessary to identify sustainable raw materials that can replace fish oil (FO) and fish meal (FM) [1,2,3].

The selection of alternative protein and lipid sources has been generally based on their composition, commercial availability, production level, current price, presence of antinutritional factors, etc. However, there is a constant need to diversify our ingredient options to provide greater resilience and allow for flexibility in formulations as the sustainability and availability of different feed ingredient sources vary depending on, for example, crop cycles, demand, and cost [4]. Testing all of these options *in vivo* is still the most robust and reliable manner to evaluate raw materials to be included in aquafeeds, but it requires the use of large numbers of animals and is time-consuming and expensive. Therefore, it would be useful to develop tools that can provide reliable indications of the nutritional profile and the possible health implications of new ingredients.

Several human and animal cellular models have been developed over recent years for investigating *in vitro* the intestinal barrier functionality [5] with the aim to generate reproducible *in vitro* models that represent the complex physiological processes that take place in the intestine as accurately as possible (for a recent extensive review see [5]). Although the vast majority of the studies have been performed in mammals, several studies have focused on fish-related models, with rainbow trout (*Oncorhynchus mykiss*) as a target species due to available cell lines. The first fish intestinal cell line, named RTgutGC, was derived from this species several years ago [6] and has been extensively studied since, for various purposes, including toxicology [7,8,9], gut immunology [10], and intestinal function [11,12,13]. 

When it comes to the study of nutrient digestion *in vitro*, the most common approach is to use species-specific enzyme extracts that mimic the stomach-to-intestine progression without cells [14,15,16]. These models can be complex and sophisticated and may be applied to investigate the mechanical and chemical breakdown of nutrients (e.g., protein hydrolysis) [17,18] but do not include the digestibility or the bioavailability of the digested nutrients by the intestinal epithelium. However, these parameters can be investigated using models that include intestinal cell lines, currently available mostly for human/mammalian studies [19,20].

To expand this option to aquaculture-relevant species, we derived and characterized two novel stable cell lines from the proximal (RTpiMI) and distal intestine (RTdiMI) of rainbow trout (RT). We demonstrated that the newly derived cell lines are composed of heterogenous call populations, retain the main characteristics of the intestinal tracts of origin, and can form an effective epithelial barrier, able to prevent paracellular flux of large molecules and absorb small molecules such as glucose and proline [21].

The aim of the present work was to combine an *in vitro* digestion protocol of fish feed for extracting a bioaccessible fraction (BAF) that does not compromise the viability of a functional intestinal epithelium so that it will be possible to evaluate both digestibility and bioavailability in the same experiment. To achieve this, we established the optimal conditions for growing functional monolayers of RTdiMI and RTpiMI cells on permeable membranes and exposed them to the bio-accessible fraction extracted from a commercially relevant fish feed. Finally, we profiled the flow through the epithelial barrier of the amino acids present in the bio-accessible fraction in different experimental conditions.

## 2. Materials and Methods

### 2.1. Propagation of Intestinal Cell Lines

We used RTpiMI and RTdiMI cell lines, previously obtained from rainbow trout proximal and distal intestines, respectively [21]. Cells were routinely cultured using Leibovitz’s L-15 medium (L15/C, Thermo–Fisher Scientific, Waltham, MA, USA cat n. 21083027) with 5% fetal bovine serum (FBS, cat. n. 10270106, Gibco, Waltham, MA, USA) and antibiotic antimycotic solution made of 100 units/mL penicillin, 0.1 mg/mL streptomycin, and 0.25 μg/mL amphotericin B (cat. No. A5955) at 20 °C in an incubator under ambient atmosphere. Cells were cultured in 75 cm^2^ tissue culture flasks (T75 cat n. 83.3911, Sarsted, Germany) and passaged at a 1:3 ratio when reaching 80% confluency. Cell passages between 30 and 35 were used for the experiments described in the present manuscript.

### 2.2. Cell Seeding and Culture on In-Well Inserts for Generating an Efficient Epithelial Barrier

Cells were seeded onto permeable polyester (PET) membrane in-well culture inserts (Greiner BioOne, Kremsmünster, Austria, ThinCert cat. No. 665640, 0.4 μm pore size, 1.13 cm^2^ surface growth area). To determine the optimal seeding density required for the formation of an effective epithelial barrier, the following seeding densities were tested: 120,000, 250,000, 500,000, and 900,000 cells/cm^2^. All experiments were performed in triplicate. Cells were cultured for 21 days in a complete medium L-15/C that was replaced twice a week. Epithelial barrier formation was checked on days 3, 7, 10, 14, and 21, measuring the transepithelial electrical resistance (TEER), evaluating the paracellular flux of Dextran, and performing a morphological analysis.

### 2.3. Measurement of Transepithelial Electrical Resistance

To assess the formation of an effective epithelial barrier, the measurement of TEER was performed using an EVOM2 Epithelial Voltohmmeter with STX2 or STX3 electrodes (World Precision Instruments, Berlin, Germany) as recommended by the manufacturer’s instructions. TEER values were obtained by subtracting the resistance values of the insert without cells from the values of the inserts measured with cells. TEER values are shown as Ω × cm^2^.

### 2.4. Paracellular Flux of Dextran

The paracellular flux of Dextran was measured as previously described [21]. Briefly, 0.6 mL of an L-15/C medium supplemented with 100 µg/mL 4 KDa FITC-Dextran (Dextran, cat. No. FD4-250MG) was pipetted in the apical compartment. Thereafter, 1.2 mL of the same medium without Dextran was pipetted in the basolateral compartment. Dextran was quantified in both apical and basolateral compartments after 6 or 24 h incubation, depending on the experiment, using a multimode microplate reader (Synergy HT, BioTek, Santa Clara, CA, USA; EX: 428 nm; EM: 540 nm) to assess the capability of the epithelial barrier to retain the molecule in the upper chamber.

### 2.5. Morphological Analysis

#### 2.5.1. Light Microscopy

For both cell lines, three culture insert membranes for each cell density were washed three times in PBS before fixation in 4% paraformaldehyde for 2 h after 21 days of culture. They were then dehydrated in a series of ethanol solutions of increasing concentration, followed by their incubation in Histoclear (National Diagnostics, Atlanta, GA, USA) for 45 min. Finally, samples were incubated in paraffin for 90 min at 60 °C before embedding. After dewaxing and re-hydration, 5–7 µm thick sections were stained with hematoxylin/eosin (HE) and observed on a Leica DMR microscope (Leica Microsystems, Milan, Italy).

#### 2.5.2. Electron Microscopy

RTdiMI cells seeded on PET membranes at densities of 70,000 cells/cm^2^ and cultured for 21 days, and 250,000 cells/cm^2^ and cultured for 7 days were prepared for electron microscopy studies. For each cell density, four membranes were prepared for scanning electron microscopy (SEM), and four membranes were prepared for transmission electron microscopy (TEM). Electron microscopy sample preparation and imaging were performed by the NMBU Imaging Centre (Ås, Norway). In brief, membranes were fixed for 2 h with freshly prepared fixation solution (1.25% glutaraldehyde, 0.1 M sodium cacodylate buffer (SCB), and 2% paraformaldehyde), with gentle agitation, washed three times for 10 min each with 0.1 M SCB and held in 0.1 M SCB at 4 °C until further processed. Samples for TEM were embedded in 3% low-melting agarose. After fixation was obtained by incubating samples for 1 h in 1% osmium tetroxide in 0.1 M SCB, samples were washed with 0.1 M SCB. Thereafter, they were dehydrated with 10 min steps in ascending ethanol series (50–100%) before proceeding with embedding in LR White resin (London Resin Company, EMS, Agar Scientific, Stansted, UK). To obtain ultrathin sections, Leica EM UC6 Ultramicrotome (Leica, Wetzlar, Germany) was used. Sections were stained for 10 min with 4% uranyl acetate and 1% potassium permanganate. Images were acquired using a Jeol JEM 2100-Plus transmission electron microscope (Jeol Ltd., Tokyo, Japan). At the same time, dehydration of the samples for SEM was performed, incubating the samples in ascending ethanol solutions (50–100%) for 10 min. Then, they were processed in a BAL-TEC Critical Point Dryer (CPD 030, Leica, Wetzlar, Germany). Coating of the samples was performed using gold/palladium with a Polaron Sputter Coater (SC 7640, Quorum Technologies, Newhaven, UK). After coating, samples were mounted on brass stubs. Images were acquired using a Zeiss EVO50 EP scanning electron microscope (Carl Zeiss AG, Oberkochen, Germany).

### 2.6. Gene Expression Analysis

The expression of the following enterocyte functional markers was analyzed in the epithelial barriers obtained using 250,000 cells/cm^2^ after 21 days of culture: leucine aminopeptidase (*lpep*) and alanine aminopeptidase (*anpep*), two proteases involved in the final digestion of peptides generated from hydrolysis of proteins by gastric and pancreatic proteases [22], and intestinal alkaline phosphatase (*iap*), a marker of terminally differentiated enterocytes [23].

RNA was extracted with TRIzol (Thermo Fisher Scientific, Waltham, MA, USA, Cat. No. 15596018) combined with PureLink RNA Mini Kit (Thermo Fisher Scientific, USA, cat. No. 12183018A), according to the manufacturer’s instructions. DNAse treatment was performed using the PureLink DNase (Thermo Fisher Scientific, USA, #12185010) as indicated in the handbook. After elution, conversion of the RNA samples to complementary DNA (cDNA) samples was obtained using an iScript Advanced cDNA Synthesis Kit for RT-qPCR (Bio-Rad, Hercules, CA, USA). To this end, samples were brought to 15 µL using nuclease-free water. Thereafter, 4 µL 5x iScript Advanced Reaction Mix and 1 µL iScript Advanced Reverse Transcriptase were pipetted to each tube containing the RNA samples. Reverse transcription (RT) was carried out by incubating the samples for 20 min at 46 °C. To inactivate the reaction, samples were incubated for 1 min at 95 °C using a DNA Thermal Cycler (Perkin Elmer, Waltham, MA, USA). Samples were stored until PCR amplification at −20 °C. Qualitative PCR was carried out using a GoTaq^®^ G2 Flexi DNA Polymerase kit (Promega Corporation, Madison, WI, USA). Each reaction was performed using 25 μL using 10.8 μL RNase-free water, 5 μL 5x Green GoTaq Flexi Buffer, 1.5 μL 25 mM MgCl, 0.5 μL 10 mM dNTPs, 5 μL 10 mM primer mix, 0.2 μL GoTaq G2 Flexi DNA polymerase, and a 2 μL cDNA sample. The program of PCR was: 95 °C for 2, 40 cycles of 95 °C for 30 s, 60 °C for 30 s and 72 °C for 5 min, and a final step at 72 ° C for 5 min. For each reaction, a tube without cDNA (negative control) was carried out for contamination exclusion. Amplicons were assessed using electrophoresis on a 2% agarose gel stained using ethidium bromide (Thermo Fisher Scientific, Waltham, MA, USA). A 50 base pair (bp) molecular marker gap (Thermo Fisher Scientific, Waltham, MA, USA) was included for each gel to verify the length of the amplicons. Since primer sequences were home-designed, expression of all the genes of the panel was also validated *in vivo*. The primers used are listed in Table S1.

### 2.7. Enzyme Activity Analysis

The activity of leucine aminopeptidase (Lpep), alanine aminopeptidase (Anpep), and intestinal alkaline phosphatase (Iap) was evaluated on the epithelial barriers of RTpi-MI and RTdi-MI.

Samples of epithelial barriers obtained using 250,000 cells/cm^2^ after 21 days of culture were fixed in formol calcium for 30 min, washed three times in double distilled water (DDW) and incubated for 1 h at room temperature and 2 h at +4 °C in a working solution of 10 mL cacodylate buffer 0.1 M pH 6.5–7.5; 0.1 mL *N*-*N*-Dimetil-formamide; Fast Blue BB Salt hemi-(zinc chloride) salt (Sigma–Aldrich, cat. No. F3378-1G); and 5 mg of L-Alanine-4-methoxy-beta-naphtylamide hydrochloride (Sigma–Aldrich, St. Louis, MO, USA, cat. No. A5414-250MG), or L-leucine Beta-naphthylamide (Sigma–Aldrich, cat. No. L1635-1G). At the end of the incubation, samples were washed thrice in DDW, incubated for 3 min in copper sulfate, and washed thrice in DDW before acquiring the images.

For alkaline phosphatase (AP) activity, cells were incubated with Tris-HCl (pH = 9.3) for 5 min to create the alkaline environment. Then, they were incubated with BCIP/NBT substrate (5-bromo-4-chloro-3-indolylphosphate/nitro blue tetrazolium) (Vector Laboratories, SK-4500, Newark, CA, USA) for 30 min at room temperature. Thereafter, they were washed three times in DDW before imaging. In both cases, images were acquired using a V16 stereomicroscope equipped with an Axiocam 506 color camera and the ZEN 2 version 2.0 package software.

### 2.8. Extraction of the Bio-Accessible Fraction (BAF) from Pelleted Fish Feed

A complete rainbow trout (RT) feed, containing 46% of crude protein and 23% of lipid level, was obtained from Skretting Aquaculture Innovation (see Table 1 for detailed composition).

The *in vitro* digestion procedure was performed based on methods reported previously with some modifications [24,25]. Briefly, ground RT feed was mixed with water (0.125 g/mL) and exposed at 20 °C to stomach (1500 U/mL pepsin) and intestinal (100 U/mL trypsin) enzyme extracts in a sequential manner simulating the gastric (pH 4, 6 h) or intestinal (pH 8, 6 h) stages, respectively. Thereafter, the *in vitro* digested feed was heat-inactivated at 90 °C for 10 min and centrifuged for 30 min at 15,000× *g*. Thereafter, the supernatant was lyophilized, resuspended in L15/ex medium (see below), sterilized using a 0.22 μm syringe filter under a horizontal laminar flow hood, aliquoted into sterile 1.7 mL tubes and stored at −20 °C until use. Crude enzyme extracts preparation as well as pepsin and trypsin activity assays were performed as described before [14]. 

### 2.9. Evaluation of the Possible Toxic Effects of the BAF on Intestinal Cells

Cells were seeded in a 96-well plate (25,000 cells/well) in 200 μL of an L15/C medium and cultured at 20 °C for 72 h prior to exposure to the digesta. For each experiment, two plates were seeded: one plate was used to estimate the number of cells by DAPI staining, and the second plate was used for a 3-(4,5-dimethylthiazol-2-yl)-2,5-diphenyltetrazolium bromide assay (MTT), which is based on the conversion of water-soluble MTT to an insoluble product (formazan) by mitochondrial reductases. Thus, higher MTT values represent a higher metabolic activity of the cells as an indicator of their viability [26] or for the combination of three fluorescent indicator dyes: Alamar Blue (resazurin), which, in alive cells, is converted to a fluorescent product by mitochondrial, microsomal, or cytoplasm oxidoreductases. Therefore, the decrease in Alamar Blue fluorescence is correlated with a decline in cellular metabolism, related to disruption of mitochondrial membranes as well [27]; CFDA-AM slowly diffuses out of intact cells since it is converted into alive cells by non-specific esterases of the plasma membrane of living cells to the fluorescent product 5-carboxyfluorescein. This product diffuses out of intact cells. Therefore, a reduction in CFDA-AM fluorescence indicates an alteration integrity of the plasma membrane [28] and Neutral Red, which measures the disruption of lysosomes resulting in a decrease in Neutral Red fluorescence [29]. All these assays can be used on the same set of cells, as previously described [13,28].

On the day of treatment, filtered BAF aliquots were thawed, used undiluted (100%), or diluted in three different concentrations (1%, 10%, and 50%) with an L15/ex medium, a simplified formulation of the L-15 containing only salts, pyruvate and galactose, but no serum [28]. After the cells were washed twice with L15/ex, the BAF dilutions or the undiluted samples were added at a final volume of 150 µL/well, and plates were put in the incubator at 20 °C for 24 h. As a control, cells were incubated for 24 h with L15/ex only. Background control wells did not contain any cells and were treated the same way as the experimental wells. At the end of the exposure to BAF, cells were washed twice with Dulbecco′s Phosphate Buffered Saline (cat n. D8662), and the following assays were performed.

For 3-(4,5-dimethylthiazol-2-yl) 2,5-diphenyltetrazolium bromide (MTT) assay, cells were washed twice with PBS and incubated with MTT Reagent (Abcam (Waltham, MA, USA) #ab211091) diluted in L15/ex and incubated for 3 h (100 μL per well). Then, the MTT reagent was carefully removed, and 150 μL of MTT Reagent Solvent was added to each well, incubated at room temperature with shaking for 15 min, and the absorbance was measured at 590 nm using a microplate reader (Synergy HT, BioTek). As a control, cells were incubated for 24 h with L15/ex only. Background control wells did not contain any cells and were treated the same way as the experimental ones. Alamar blue (AB), CFDA-AM, and Neutral Red assays were performed as described previously [26,27]. For DAPI staining, following the exposure of the cells to digesta for 24 h, the cells were washed twice with PBS, fixed with 4% PFA for 10 min at room temperature, washed again with PBS, and incubated with DAPI (300 nM) for 5 min. Cells were then washed with PBS, and fluorescence was measured using a multimode microplate reader (Synergy HT, BioTek). Background control wells did not contain any cells and were treated the same way as the experimental ones.

### 2.10. In Vitro Cell Assays

We performed preliminary tests to determine the more suitable medium to resuspend the BAF and to quantify the amount of single amino acids present in the samples before and after their exposure to the cell lines. As described in detail in Appendix A, it was necessary to eliminate the amino acids contained in the complete culture medium used for propagating the cell lines; therefore, we used an L15-modified medium (L-15/ex, [28]). We also determined that the presence of 5% fetal bovine serum would prevent accurate measurement of the amino acid profile but found that the presence of 0.4% (*w*/*v*) of bovine serum albumin (BSA) was tolerated by the analytical procedure provided that samples were centrifuged at 10,000× *g* for 10 min and filtered through a 10 kDa MWCO Amicon filter (cat. n. Z648027-24EA). It was necessary to add at least 25% BAF to enable an accurate quantification. Therefore, the following eight (2 × 2 × 2) combinations were tested on the cell layers: 50% and 25% (*v/v*) of BAF diluted in L-15/ex, with or without BSA, and with an exposure time of either 6 or 24 h. All combinations were spiked with 100 µg/mL 4 KDa FITC-Dextran. In total, 0.5 mL of each media was pipetted into the upper compartment of the inserts, and 1.2 mL of L15/ex was pipetted into the lower compartment. After 6 and 24 h of exposure, a sample of 0.1 mL samples was collected from the upper and lower compartments of the inserts. The quantification of FITC-Dextran was evaluated by means of a multimode microplate reader (Synergy HT, BioTek) to analyze the epithelial barrier integrity during the experiment. Afterward, epithelial barriers were fixed by a mean of 4% paraformaldehyde. Then, hematoxylin and eosin staining were performed to assess cell morphology. At the same time, endogenous alkaline phosphatase (AP) activity was performed to identify fully differentiated enterocytes. For these experiments, epithelial barriers were developed by seeding RTpiMI and RTdiMI cells at a density of 250,000 cells/cm^2^ and culturing for seven days, as described above.

Exposure to the bio-accessible fraction was performed after the TEER value reached its plateau (i.e., after two consecutive measurements carried out at 3, 7, and 10 days gave similar values), and the paracellular flux of Dextran significantly dropped. The same parameters were measured at the end of the exposure to confirm that no damage had occurred. Moreover, to confirm the absence of a negative effect, cell morphology was evaluated with hematoxylin and eosin staining.

### 2.11. Biochemical Analysis

Amino acid (AA) quantification of triplicate samples, collected from the apical and compartments of epithelial barriers obtained using 250,000 cells/cm^2^ and cultured for seven days, was performed by high-performance liquid chromatography coupled with Exploris-Orbitrap^®^ high-resolution mass spectrometry (HPLC-Exploris-HRMS) (Thermo Fisher Scientific, San Jose, CA, USA). Chromatographic separation of AA was accomplished on Vanquish HPLC instrument using Thermo Scientific™ Accucore™-150-Amide-HILIC column (2.1 × 150 mm, 2.6 µm) with the gradient of two mobile phases (acetonitrile and water both enriched with 20 mM ammonium formate). An Exploris-HRMS mass spectrometer operated in both positive and negative ionization modes was performed with predetermined acquisition parameters. The full scan was used with resolving power 120,000 (scan range of *m*/*z* 70–350) for the screening and statistical evaluation of the chromatographic profiles. Full scan data-dependent acquisition (FS-dd-MS2) with resolving power 60,000 and 30,000 for full scan and dd-MS2, respectively, was performed for fragmentation of pseudo-molecular ions detected in FS mode. Fragmentation of precursors was done with stepped, normalized collision energy (NCE) set at 20 eV and 30 eV.

The following 14 amino acids were quantified: L-Alanine, L-Isoleucine, L-Leucine, L-Methionine, L-Phenylalanine, L-Tryptophan, L-Valine (all non-polar AA); L-Arginine, L-Lysine (both basic AA); L-Asparagine, L-Glutamine, L-Threonine, and L-Tyrosine (polar AA). The quantity of each amino acid in the upper and lower compartments was compared to the BAF sample added to the cells at the beginning of the experiments (BAF_t0_).

The mass balance of each amino acid was calculated with the formula:

Mass Balance = (A + BL) − BAF_t0_

where A is the amino acid amount in the apical compartment, BL is the amino acid amount in the basolateral compartment, and BAF_t0_ is the amino acid amount contained in the BAF used for all experiments and added to the system at time 0. A negative figure indicates a loss, and a positive figure indicates a gain.

The transport across the epithelial barrier of each amino acid was calculated as the percentage of the amino acid quantified in the lower compartment after 6 and 24 h incubation compared to the initial amount pipetted in the apical compartment, calculated as follows:

Transport = BL/(A + BL) × 100.

A control sample, represented by the membrane without any cells, indicated as NC was run in triplicate for each treatment.

### 2.12. Statistical Analysis

Unless otherwise stated, results are shown as mean ± standard deviation calculated from three replicates. Analysis was run using one-way ANOVA. Multiple comparisons were performed using Tukey’s post hoc tests using PRISM version 8.2.1 (GraphPad Software, San Diego, CA, USA). The statistical significance of the results was considered when *p* < 0.05.

When appropriate, the Pearson Chi-square and results were considered statistically significant when *p* < 0.05.

## 3. Results

### 3.1. Development of a Functional Epithelial Barrier on PET Membranes

**Figure 1:**
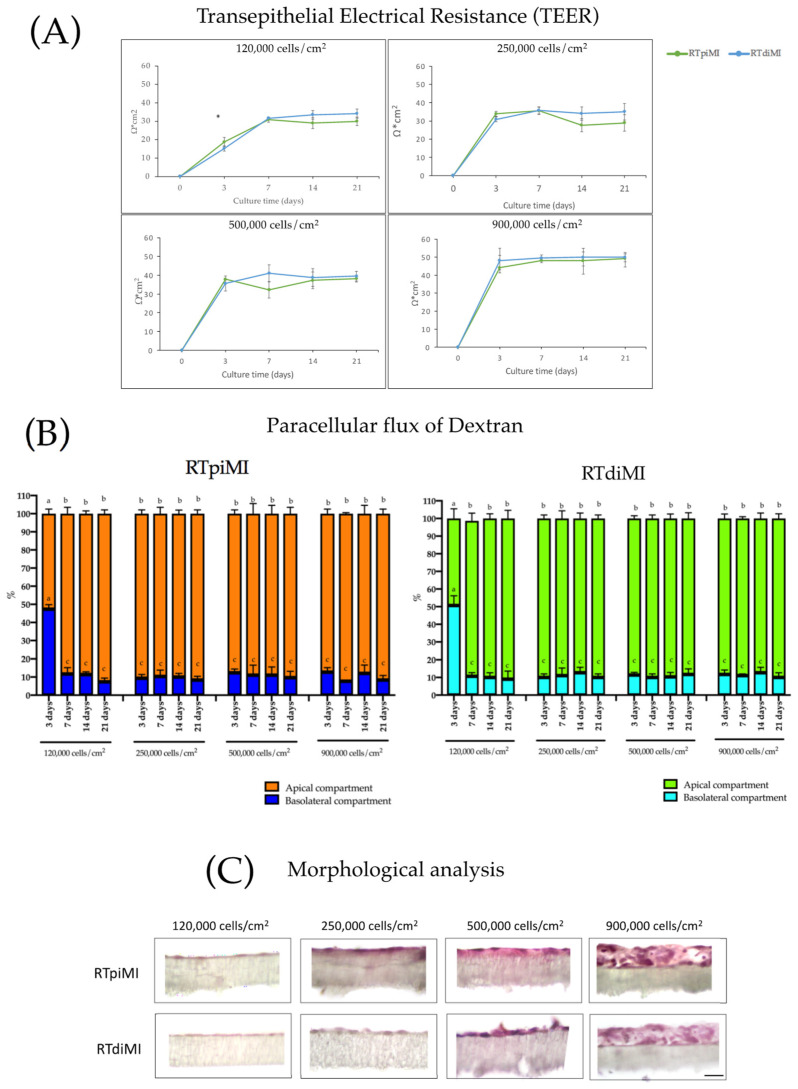
Development of RTpiMI and RTdiMi epithelial barriers. Measurement of TEER (**A**) and dextran paracellular flux. Asterisk (*) indicates statistically significant differences within the groups (*p* value < 0.05). (**B**) at 3, 7, 14, and 21 days of culture with the following seeding density: 120,000, 250,000, 500,000, and 900,000 cells/cm^2^. Different lower case letters indicates statistically significant differences within the groups (*p* value < 0.05). (**C**) Morphological evaluation of epithelial barrier obtained with 120,000, 250,000, 500,000, and 900,000 cells/cm^2^ cultured for 21 days. Scale bar = 50 µm.

We investigated how long it takes RTpiMI and RTdiMI lines to form a functional epithelial barrier. We tested the 120,000, 250,000, 500,000, and 900,000 cells/cm^2^ seeding densities. At 120,000 cells/cm^2^, both cell lines formed an effective epithelial barrier at seven days of culture, as indicated by TEER, whose value reached a plateau at day 10 (Figure 1A). Increasing the seeding density, a functional barrier was obtained already at three days of culture for both cell lines (Figure 1A) and the plateau was reached at day seven, as indicated by the same parameter. No differences were observed above 250,000 cells/cm^2^. In line with these results, when TEER reached its plateau, the epithelial barriers formed by both cell lines significantly increased (*p* < 0.05) the cells’ ability to retain 4 kDa Dextran in the apical compartment, demonstrating the ability to inhibit the paracellular flux of large molecules (Figure 1B).

**Figure 2:**
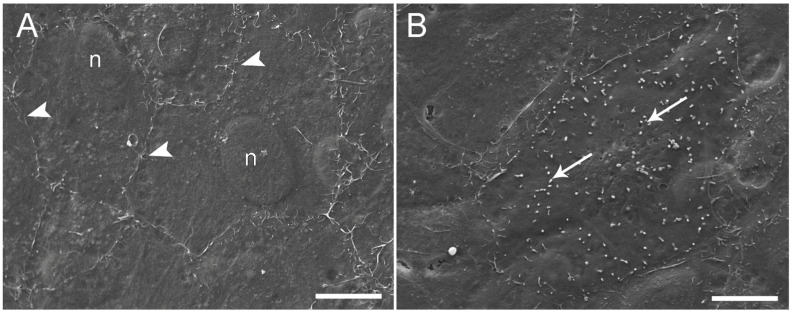
Scanning electron microscopy of RTdiMI cells grown on PET membranes. (**A**) Coherent cell layer with thread-like structures at cell borders (white arrowheads). (**B**) Cell surface covered by possible microvillus-like structures (arrows). n: nuclei. Scale bars 10 µm.

**Figure 3:**
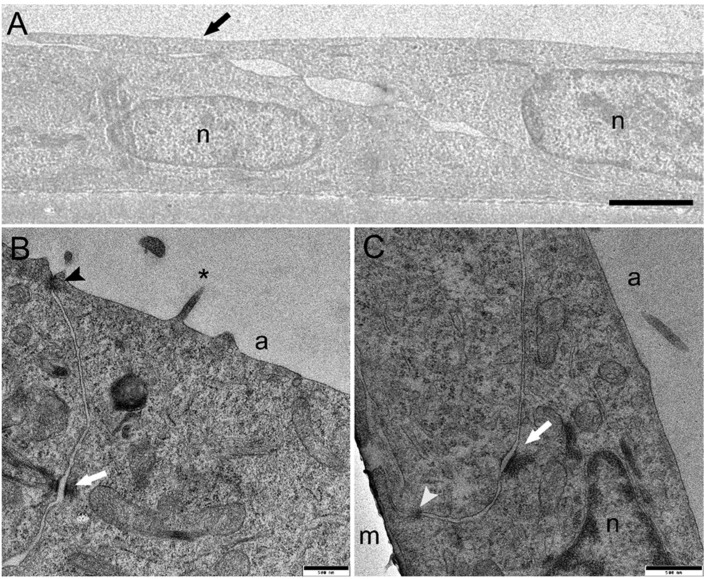
Transmission electron microscopy of RTdiMI cells grown on PET membranes. (**A**) TEM confirmed a monolayered cellular barrier. The black arrow indicates the border between cells by extending the apical arm or thread. (**B**) Apical tight junction (black arrowhead) and lateral desmosome (white arrow). Asterisk indicates protrusion from the cell surface. (**C**) Lateral desmosome (white arrow) and basal gap junction (white arrowhead). n: nuclei, a: apical side, m: membrane. Scale bars (**A**) 1.5 µm, (**B**) and (**C**) 500 nm.

Morphological analysis by light microscopy performed after 21 days of culture revealed that at a seeding density of 120,000 and 250,000 cells/cm^2^, both cell lines formed a monolayer on the membrane (Figure 1C). In comparison, higher seeding density determined the formation of stratified cell layers (Figure 1C). These results indicated that 250,000 cells/cm^2^ was the optimal seeding density to obtain an effective epithelial barrier formed by a cell monolayer with a culture time shorter than that used in previous work; thereby, the cell inserts exposed to BAF were prepared using this protocol. Electron microscopy studies of RTdiMI cells confirmed a coherent and polarized monolayer by a seeding density of 250,000 cells/cm^2^ and 7 days culture in similarity to a seeding density of 70,000 cells/cm^2^ and 21 days culture (Figure 2 and Figure 3). SEM imaging revealed coherence of the cell layer with thread-like structures at the cell borders appearing to stitch the cells together and occasional cells with small protrusions on the apical surface (Figure 2). TEM studies indicated structural polarization of the epithelium by apical tight junctions, lateral desmosomes, and basal gap junctions (Figure 3).

### 3.2. Differentiation of Functional Enterocytes, Forming Epithelial Barriers

**Figure 4:**
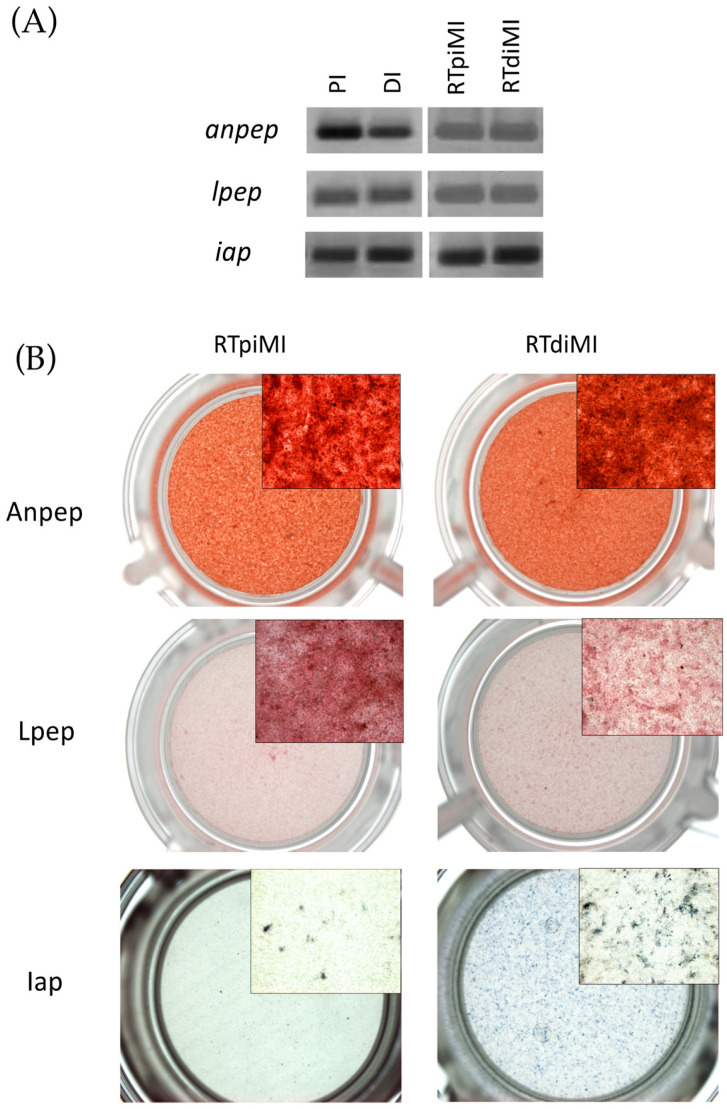
Gene expression and enzyme activity. (**A**) Expression of leucine aminopeptidase (*lpep*), alanine aminopeptidase (*anpep*), and intestinal alkaline phosphatase (*iap*) transcripts of rainbow trout proximal (PI) and distal (DI) intestine, and RTpi-MI and RTdi-MI barriers. (**B**) Analysis of enzyme activity of leucine aminopeptidase (Lpep), alanine aminopeptidase (Anpep), and intestinal alkaline phosphatase (Iap) in RTpi-MI and RTdi-MI barriers.

Firstly, we validated the specificity of the primer sequences that we designed, examining the gene expression in the *in vivo* proximal (PI) and distal (PI) intestine (Figure 4A). Thereafter, we found that RTpiMI and RTdiMI cells express alanine and leucine aminopeptidases and intestinal alkaline phosphatase (Figure 4A), which are functional markers of fully differentiated enterocytes.

Differentiation was further indicated by the detection of alanine and leucine aminopeptidases activity in both cell lines at the same level (Figure 4B). Alkaline phosphatase activity, however, was higher in RTdiMI than in RTpiMI cells (Figure 4B).

### 3.3. Identification of Concentrations Compatible with Intestinal Cell Viability

The possible cytotoxic effect of the BAF extracted from the feed pellets was evaluated by exposing the cells for 24 h to the undiluted sample or after its 1%, 10%, and 50% dilution in the L15/ex medium.

**Figure 5:**
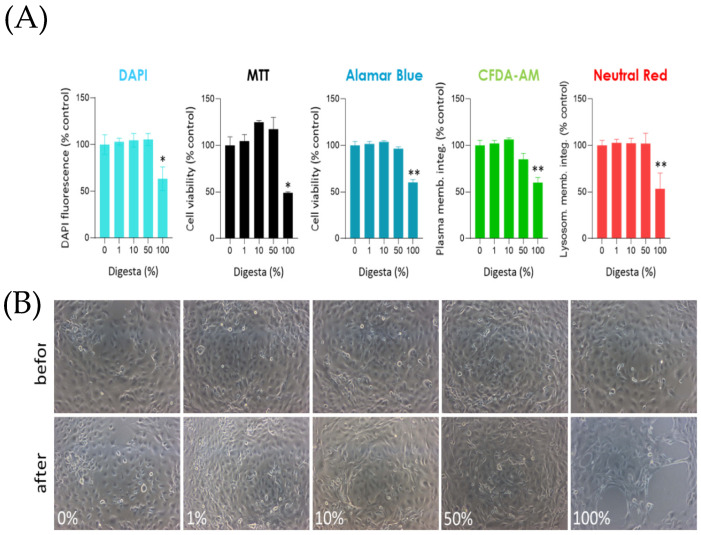
Cells were seeded in 96-well plates and, after 72 h, were exposed to different BAF concentrations (1%, 10%, 50%, and 100%) for 24 h. (**A**) Double-stranded DNA was fluorescently labeled with DAPI to indirectly estimate cell proliferation after treatments. Other assays evaluated metabolic status (MTT and Alamar Blue), plasma membrane integrity (CFDA-AM), and lysosomal integrity (Neutral red). Results are expressed as mean ± SE of at least three independent experiments for each assay. In all the assays, significant differences were found only when cells were exposed to undiluted BAF (100%) with respect to the control (0%). (**B**) Representative micrographs of cells before and after the 24 h treatment with digesta. Data were analyzed with a one-way ANOVA followed by Tukey’s post-hoc test (* *p* < 0.05, ** *p* < 0.01).

DNA labeling with DAPI showed a sharp decrease (*p* = 0.001) when the undiluted fraction (100%) was used (Figure 5A), caused by a notable reduction of cells attached to the well (Figure 5B). The treatment with other concentrations (1%, 10%, and 50%) did not cause any significant reduction of DAPI fluorescence, indicating that the BAF had no effect, consistent with what was found with the other assays performed after the treatments. In fact, 100% BAF induced a significant decrease of MTT (*p* = 0.01), Alamar Blue (*p* = 0.01), CFDA-AM (*p* = 0.0013), and Neutral Red (*p* = 0.001) signals, whereas the other BAF concentrations did not (Figure 5A). Figure 5B shows representative micrographs of RTdi-MI cells before and after the treatment with different concentrations. As observed, the treatment with the undiluted sample caused a severe loss of cells attached to the well, which explains the significantly lower DAPI signal. At 50% concentration, a slight change was observed in the morphology of the cell layer; however, the cell viability assays did not show any significant effect (see Figure 5B). Finally, no changes were observed when 1% or 10% concentrations were used, consistent with the cytotoxicity assays.

Taken together, these results suggest that up to 50% concentration in L15/ex does not cause any significant detrimental or cytotoxic effect on the metabolic status, cell plasma membrane integrity, or lysosomal integrity of intestinal epithelial cells.

### 3.4. Exposure of Intestinal Epithelial Barrier to the BAF Extracted from the Pelleted Feed

#### 3.4.1. Analysis of Barrier Integrity after Exposure to BAF

**Figure 6:**
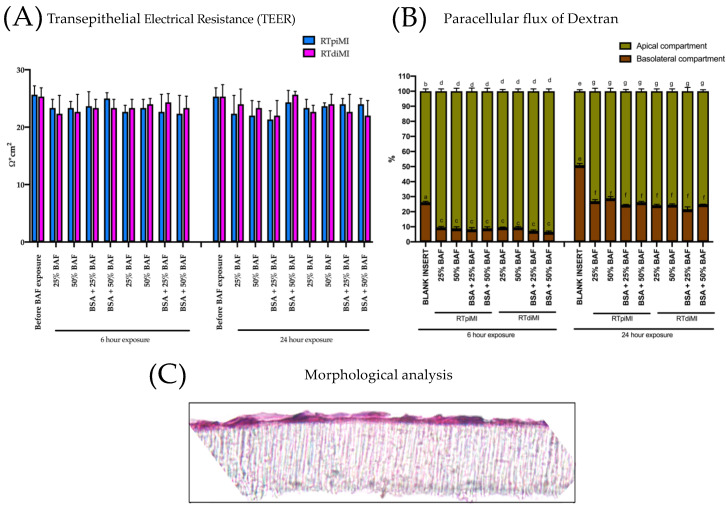
Barrier integrity at the end of the exposure to different BAF concentrations in different media for different lengths of time. (**A**) TEER did not significantly change in all treatments at the end of the experiments. (**B**) 4 KDa Dextran remained in the upper chamber due to the intact morphology of the epithelial monolayer represented in panel (**C**). Different lower case letters indicate statistical significance (*p* value < 0.05).

Following the preliminary experiments described in Appendix A, RTpiMI and RTdiMI cells were exposed to 25% and 50% BAF diluted in an L-15/ex medium with and without BSA for 6 and 24 h. The integrity of the epithelial barrier was not affected in both cell lines, as indicated by the constant TEER values, and after all treatments, both RTpi-MI and RTdi-MI cell barriers retained Dextran 4 KDa in the apical compartment of the inserts at a significantly higher rate (*p* < 0.05) than the inserts without cells (Figure 6A,B). The morphological analysis confirmed the presence of an intact cell monolayer on the membrane, further confirming that IVD incubation did not alter the epithelial barrier functionality after 6 or 24 h incubations (Figure 6C).

#### 3.4.2. Profiling the Amino Acid Content in the Different Experimental Conditions

As described in detail, we performed a preliminary trial limited to 4 AA to identify the experimental conditions that would enable the observation of the fate of each of the 14 AA analyzed in the full experiment.

Table S2 shows that it was possible to quantify the concentration of all 14 AAs examined in the BAF in all the experimental conditions (with and without BSA, at 25% and 50% dilution) and at all time points (0, 6, and 24 h). All measurements have been performed in the presence of both cell lines (RTpiMI and RTdiMI) as well as without cells. All experiments were run in triplicate.

Since the experiments were performed with a single diet, all 14 AAs were grouped in order to detect possible effects exerted by the different combinations of experimental parameters.

The recovery rate at the end of the exposure time was determined to detect if some of the AA were used directly by the cells (Table 2). After 6 h of exposure, the amount of some of the 14 amino acids (AA) measured in the BAF was increased compared to the initial sample. Significant differences (*p* < 0.05) were measured in the presence of BSA with 50% BAF and in the absence of BSA with 25% BAF. After 24 h, the phenomenon almost reached the plateau since the amount of 12 of the 14 AAs analyzed in the BAF was significantly increased (*p* < 0.05) at both 25% and 50% concentration compared to the quantity measured at time 0. The presence or the absence of BSA in the medium and the presence or absence of cells did not have an impact at this time point.

The amount of AA that moved from the apical to the basolateral compartment was measured. However, since the determination of the total amount of AA increased during the experiments, in particular after the 24 h exposure, we expressed the amount of each AA found in the basolateral compartment as the percentage of the total amount found in the system. This enabled us to compare the different amino acids and to group them in different ways. As presented in Table S2, the flow pattern of each amino acid does not seem to be influenced by any of the variables that were studied (BAF concentration, presence of BSA time of exposure, or cell line). Therefore, we considered the AA as a group, and we examined the effect of the presence of the cells and of the origin of the cell line (proximal or distal intestine). When the BAF was dissolved in a medium with BSA, the presence of cells and the cell type had an unpredictable role in the flux of amino acids from the apical to the basolateral compartment. In the absence of BSA, both proximal and distal intestinal cells slowed the amino acids flux at 50% BAF concentration, but only RTdiMI cells did the same at 25%. In all cases, the time of exposure did not have a significant effect (Table 3A).

When we considered the chemical nature of the amino acids, the results indicate that irrespective of the cell line, non-polar AAs can move from the apical to the basolateral compartment less efficiently than polar and basic AAs, with the only exception when cells are exposed to BAF for 24 h diluted in medium with no BSA (Table 3B).

## 4. Discussion

In the present paper, we combined the *in vitro* digestion of feed pellets with the exposure of the resulting product to intestinal cell lines to develop an *in vitro* platform that can evaluate both digestibility and absorption. Our main aim was to determine the experimental conditions that would best enable the combination of these approaches for gathering physiologically relevant data.

As recommended for the development of a standardized test, it is important to determine the conditions when the test itself can be performed in a repeatable way [19]. Therefore, it was essential to determine when a functional epithelium is formed through the attainment of the TEER plateau and the ability to retain larger molecules in the upper chamber. Since speed and high throughput capacity would be important for the commercial application of this test, it has been important to determine that increasing the seeding density from ~70,000 to 250,000 cells/cm^2^ enabled the formation of a functional barrier in three rather than in 21 days [11,21] and polarization of the monolayer by apical positioning of tight junctions after 7 days. Even more important was the observation that using higher seeding density determined the formation of stratified cell layers that would significantly alter the resemblance with the *in vivo* intestinal epithelium. It is interesting to note that at the highest density, both the number of layers and the TEER values were highest, indicating that TEER is a sensitive parameter for evaluating not only barrier integrity but also cell proliferation. Barrier formation was also accompanied by the expression of brush border enzymes and by the detection of their activity. This confirmed our previous observations indicating that increasing the seeding density stimulates cells to differentiate [21]. Furthermore, such differentiation, at the appropriate seeding density (250,000 cells/cm^2^), was accompanied by a decrease in cell proliferation as indicated by the presence of a monolayer for at least 21 days.

The combination of an *in vitro* digestion based on the use of fish enzymes working at 20 °C with exposure to intestinal cells *in vitro* was possible because the process produced a chyme-like fraction that did not damage the cell monolayers. This was obtained with sequential exposure to gastric and intestinal enzyme extracts followed by their thorough inactivation. The removal of any particulate matter and filter sterilization ensured its compatibility with the cells. This could not be taken for granted because, although the markers for goblet cells were found in both cell lines [21], the main cell type present in the culture was enterocytes. Therefore, these cells cannot take advantage of the full protective elements provided *in vivo* by the goblet cells in the form of an effective mucus layer, the first line of defense against microorganisms, digestive enzymes and acids, digested food particles, and food-associated toxins [30]. Nevertheless, our data showed that the system has a large operative margin since it can tolerate, without apparent negative effects, concentrations as high as 50%. At present, we have not identified the reason for the toxic effect of undiluted BAF. 

The numerous efforts to compare *in vivo* and *in vitro* digestion systems are mostly focused on models without cells [31]. However, the need for coupling *in vitro* food digestion with *in vitro* epithelial absorption is becoming more evident and is attracting increasing efforts, almost exclusively in human models [32]. The main aim of our experiments was to develop a system that would enable to profile amino acid transit through a reconstructed intestinal epithelial barrier following its exposure to a physiologically relevant BAF of a commercially meaningful feed in fish. Therefore, it was important to define the experimental conditions that would enable accurate quantification of AA within a complex mixture.

In this respect, we achieved our goal with the identification of BAF extraction and exposure conditions that allow precise quantification of the AAs using high-resolution mass spectrometry. It was soon evident that the number of AAs present in the standard L-15 medium caused a background noise that prevented the accurate AA quantification present in the BAF. The problem was solved with the use of the L-15/ex medium that has no AA in its composition. We tested the medium both with and without BSA looking for the best compromise between accurate AA quantification and full cellular function. The presence of BSA in the culture medium, in fact, has several positive functions binding and stabilizing many important small molecules and ions, is an antioxidant, and binds fatty acids, copper, and other important molecules [33]. At the same time, it is less prone to batch variations than serum and adds fewer undefined variables. The results indicate that the presence of BSA in some circumstances affects the results, but there have been no signs that it compromised the possibility of an accurate AA quantification. Overall, these experiments showed that it is important to select a medium that combines well with the analytical technique used downstream.

It was interesting to note that the level of AAs increased during the experiment (Table 2). While after 24 h, the process had already reached its plateau, and no differences were visible among treatments, the data after 6 h were more informative. First, it was evident that, although the increase was visible even in the absence of cells, it was significantly more pronounced when cells were present. This suggests that both cell lines have the capacity to cleave small peptides and is consistent with the peptidase activity found in both cell lines, confirming previous observations [21]. However, the fact that after 24 h, the increase involved most AAs, even without cells in the system, implies the presence of some residual proteolytic activity in the BAF. This indicates that there is room for improving the enzyme inactivation procedures, and this parameter must be checked as a matter of routine for standardizing the procedures.

While it has been possible to quantify each AA in all treatments, no clear pattern has emerged, also in consideration that we did not perform a comparison between two or more feeds. Therefore, we looked at the AAs as a group, and we considered the effect of the cell line (Table 3A). We observed that in the absence of BSA and at 25% dilution, proximal cells are more efficient than distal cells in translocating AAs from the apical to the basolateral compartment. This is consistent with the fact that most AAs are absorbed in the proximal intestine [34] and confirms that RTpiMI and RTdiMI lines preserve some characteristics of their intestinal tract of origin. Differences between cell lines were no longer evident when 50% BAF concentration was used, possibly due to a saturation of the transporters.

In fish enterocytes, the SLC15A1 peptide transporter, better known as PEPT1, is responsible for the absorption of approximately 80% of digested proteins in the form of tri- and di-peptides [35,36]. Separate transporters handle the transit of neutral, basic, acidic, and non-polar AAs mostly across the basolateral membrane [37]. Therefore, we grouped the AAs based on their charge, and we noticed that in most circumstances, non-polar AAs are less efficiently transported through the *in vitro* epithelial barrier than polar and basic AAs. No acidic AAs were present among those quantified in this study. The SLC1A5 transporter is responsible for the transit of non-polar AAs that include alanine, serine, cysteine, threonine, glutamine, asparagine, methionine, glycine, and leucine [38], and it will be interesting to quantify its presence in future studies.

## 5. Conclusions

To the best of our knowledge, this is the first attempt to develop a platform that combines the *in vitro* digestion of pelleted feed with the exposure of intestinal epithelial cells to the extracted bio-available fraction (BAF) to determine amino acid digestibility and bioavailability at the same time in fish. We achieved the main aim of developing the techniques necessary for combining the two approaches and showed that it is possible to quantify the amount of any single amino acid during the procedure. We found that BAF concentration, the medium used for its dilution, the length of exposure, and the different epithelial cell lines, can all influence the results and, therefore, must be selected according to the final aim of the experiment.

On this basis, it will be interesting to compare different feeds and explore how the results that will be obtained can be correlated with the information gathered from *in vivo* trials.

## Figures and Tables

**Table 1:** Composition of the feed pellets is expressed on “as is” basis.

Ingredient	Control
Corn gluten	5.6
Wheat gluten	15.5
Faba bean meal	7.9
Sunflower meal	10.4
Wheat	9.0
Fish oil	8.0
Fish meal	34.9
Rapeseed oil	8.2
Premixes; vit, min, astaxanthin	0.7
Nutrient Volume %	100.0
Moisture, %	7.0
Crude Protein, %	46.5
Crude fat, %	23.0
Starch, %	10.0
Ash, %	6.2
DE MJ/kg	19.9
DP g/kg	413.0
Crude Fiber, %	0.4
NFE, %	16.9

**Table 2:** Number of amino acids whose concentration was higher at the end of the exposure compared to their initial amount as calculated with the Mass Balance formula. Six and twenty-four hours are the length of the exposure. BSA = 4 mg/mL BSA in the medium. noBSA = 0 mg/mL BSA in the medium. The 25 and 50 = BAF concentration in the medium. RTpiMI proximal intestine cell line; RTdiMI distal intestine cell line. Different superscripts in the same row indicate a significant difference between groups (*p* < 0.05).

		+BSA				−BSA			
		6 h		24 h		6 h		24 h	
	BAF (%)	25	50	25	50	25	50	25	50
Cell Lines									
RTpiMI		6	3 ^a^	12	12	9 ^a^	7	12	12
RTdiMI		8	6 ^a^	12	12	10 ^a^	8	12	12
No cells		3	1 ^b^	12	11	1 ^b^	6	12	12

**Table 3:** (**A**) Average rate of amino acids that moved from the apical to the basolateral compartment of the culture insert in relation to the cell line. NC = no cells; Pi = RTpiMI proximal intestine cell line; Di = RTdiMI distal intestine cell line. (**B**) Average rate of amino acids that moved from the apical to the basolateral compartment of the culture insert in relation to the AA type PO = polar; NP = non-polar; BA = basal amino acids. L-15/ex BSA = medium supplemented with 4 mg/mL BSA. L-15/EX no-BSA = 0 mg/mL BSA in the medium. The 25% and 50% = IVD concentration in the medium. Different superscripts in the same row indicate significant differences between groups (*p* < 0.05).

**(A)**							
	**6 h Incubation**			**24 h Incubation**			
50% BAF	48.0 ^a^	48.7 ^a^	46.9 ^a^	59.6 ^b^	45.4 ^c^	64.7 ^a^	**L-15/ex with BSA**
25% BAF	60.6 ^a^	50.4 ^b^	44.6 ^c^	47.9 ^a^	45.2 ^b^	45.7 ^b^	
**Cell lines**	**NC**	**Pi**	**Di**	**NC**	**Pi**	**Di**	
50% BAF	62.9 ^a^	48.0 ^b^	44.7 ^b^	59.3 ^a^	47.2 ^b^	45.5 ^b^	**L-15/ex without BSA**
25% BAF	53.1 ^a^	47.7 ^a^	33.2 ^b^	50.2 ^a^	50.4 ^a^	47.7 ^b^	
	**6 h incubation**			**24 h incubation**			
**(B)**							
	**6 h Incubation**			**24 h Incubation**			
50% BAF	50.4 ^a^	45.3 ^b^	51.1 ^a^	57.9 ^a^	55.3 ^b^	57.4 ^ab^	**L-15/ex with BSA**
25% BAF	56.3 ^a^	49.5 ^b^	53.4 ^a^	47.9 ^a^	44.6 ^b^	48.1 ^a^	
**AA type**	**PO**	**NP**	**BA**	**PO**	**NP**	**BA**	
50% BAF	54.1 ^a^	50.3 ^b^	52.5 ^ab^	51.4 ^a^	50.6 ^a^	49.8 ^a^	**L-15/ex without BSA**
25% BAF	44.6 ^a^	41.2 ^b^	51.4 ^a^	49.0 ^b^	48.2 ^b^	52.8 ^a^	
	**6 h incubation**			**24 h incubation**			

## Data Availability

The data presented in this study are openly available in the Fish-AI repository hosted at the URL: “https://dataverse.unimi.it/dataverse/fish-AI”.

## Supplementary Materials

The following supporting information can be downloaded at: https://www.mdpi.com/article/10.3390/ani13142278/s1, Table S1: primer sequences; Table S2: amino acids quantification.

## Appendix A

Determination of optimal dilution medium, BAF concentration, and length of exposure.

In a series of preliminary experiments, we measured the L-Alanine, L-Arginine, L-Histidine, and L-Phenylalanine concentrations to determine the optimal dilution medium and BAF concentration that would enable for the most accurate ammino acid quantification allowed by the instrument sensitivity. The four amino acids were quantified as described above, dissolving the lyophilized BAF in (i) L-15 complete medium with 5% FCS; (ii) L-15/ex with 4 mg/mL BSA; (iii) L-15/ex without BSA at 1%, 10%, 25%, or 50% (*v/v*) concentration. The results summarised in Table A1 showed that the background noise prevented an accurate amino acid quantification when BAF was diluted in l/15 complete medium with 5% in any concentration. On the other hand, quantification of all amino acids was possible when BAF was diluted in L-15/ex both with and without BSA but only when using 25% and 50% of IVD dilution (Table 1). Therefore, all the experiments were run using 25% and 50% IVD in L/15/fx with and without BSA.

**Table A1:** Quantification of L-alanine, L-arginine, L-histidine, and L-phenylalanine in different media combinations. Results are shown as mg/mL.

Medium	L-Alanine	L-Arginine	L-Histidine	L-Phenylalanine
L-15	225.0	500.0	250.0	125.0
L-15 + BSA	223.0	497.0	255.0	119.0
L-15/ex	N.D.	N.D.	N.D.	N.D.
L-15/ex + BSA	N.D.	N.D.	N.D.	N.D.
Undiluted BAF (100%)	178.7	231.9	36.8	108.3
L-15 + BSA + 1% IVD	206.9	483.9	219.6	121.5
L-15 + BSA + 10% IVD	209.8	485.6	228.2	109.4
L-15 + BSA + 25% IVD	210.9	485.6	194.0	98.0
L-15 + BSA + 50% IVD	178.7	382.0	197.8	97.3
L-15/ex + 1% IVD	N.D.	3.1	0.7	1.5
L-15/ex + 10% IVD	13.6	23.7	4.9	13.2
L-15/ex + 25% IVD	34.9	67.8	11.3	29.4
L-15/ex + 50% IVD	75.7	134.4	20.2	54.2
L-15/ex + BSA + 1% IVD	N.D.	2.2	0.9	1.0
L-15/ex + BSA + 10% IVD	N.D.	15.3	2.5	9.7
L-15/ex + BSA + 25% IVD	23.3	39.3	5.4	21.2
L-15/ex + BSA + 50% IVD	38.0	99.4	12.0	42.4

In order to determine for how long we could expose the cells to the 25% and 50% IVD dissolved in L-15/ex with and without BSA, we evaluated the barrier integrity after 6 and 24 h in all possible combinations measuring the TEER values and the concentration of FITC-Dextran 4 KDa in the apical compartment. The constant TEER values and the ability of both RTpi-MI and RTdi-MI cell barriers to retain FITC-Dextran 4 KDa in the apical compartment at a significantly higher rate than the inserts without cells indicated that both exposure times would provide reliable results. In agreement with these results, after all treatments, morphological analysis confirmed the presence of an intact cell monolayer on the membrane, further confirming that IVD incubation altered the epithelial barrier functionality neither after 6 nor 24 h incubations to both 25% and 50% BAF concentrations dissolved in L-15/ex with and without 4 mg BSA/mL. These six combinations were used for the rest of the experiments.
