# Supplementary material for: Development of a Rainbow Trout (Oncorhynchus mykiss) Intestinal In Vitro Platform for Profiling Amino Acid Digestion and Absorption of a Complete Diet
Source: Animals (Basel). 2023 Jul 12;13(14):2278. doi: 10.3390/ani13142278 (PMC10376269; doi:10.3390/ani13142278)
Supplement: Supplementary file 1 [file animals-13-02278-s001.zip › animals-2468543-supplementary.pdf]

Table S1. List of primer sequences used for gene expression analysis. Gene ID, amplicon size in base pairs (bp), accession number ID, forward and reverse primer sequences, annealing temperature are reported for each gene.

Gene ID (amplicon size in base pairs (bp))	Accession Number ID	Forward (F) and reverse (R) Primer Sequence (5->3)	Annealing Temperature (°C)	Reference
lpep (100 bp)	XM_036972002.1	F: CTTTGACAGTGGTGGGATCA<br>R: TGTGACGATGGCGGAAC	60	Own Design
anpep (139 pb)	XM_021568270.2	F: GGAATGACCTGTGGCTGAAT<br>R: GCCAACGAATCCACTTGAAAC	60	Own Design
iap (157 bp)	XM_021606337	F: TTGGCACGGAAAGAGAGTCC<br>R: CATGGGAAAGCTACCAGGCA	60	Own Design

Table S2. *a:* Amino acid amount (µg) detected in the apical (AP) and basolateral (BL) compartment of ThinCert inserts after **6 hours** of exposure to bio-available fraction (BAF) in the different culturing conditions. RTdiMI (RTDI) and RTpiMI (RTPI) cell lines were exposed to 25% or 50% BAF diluted in L15/ex medium supplemented with 4% BSA or without any supplementation (no BSA). The same conditions were also used on control samples without cells (NC).

BSA	BSA	BSA	BSA	BSA	BSA	BSA	No BSA	No BSA	No BSA	No BSA	No BSA	No BSA	BSA	BSA	BSA	BSA	BSA	BSA	No BSA	No BSA	No BSA	No BSA	No BSA	No BSA
%BAF	25	25	25	25	25	25	25	25	25	25	25	25	50	50	50	50	50	50	50	50	50	50	50	50
Cells	NC	RTDI	RTPI	NC	RTDI	RTPI	NC	RTDI	RTPI	NC	RTDI	RTPI	NC	RTDI	RTPI	NC	RTDI	RTPI	NC	RTDI	RTPI	NC	RTDI	RTPI
Compartment	AP	AP	AP	BL	BL	BL	AP	AP	AP	BL	BL	BL	AP	AP	AP	BL	BL	BL	AP	AP	AP	BL	BL	BL
	7,90	18,28	15,28	11,96	17,13	14,10	6,65	16,68	12,69	9,64	17,13	14,10	11,15	27,59	26,64	17,83	28,71	24,56	18,34	28,18	23,99	34,82	13,55	23,96
L-Alanine	8,00	16,61	14,35	13,50	15,48	13,54	7,00	19,79	10,93	10,00	15,48	13,55	12,50	27,42	27,68	18,00	29,55	25,78	20,00	26,51	25,16	37,00	12,96	25,38
	7,95	16,87	13,67	12,73	14,10	14,56	6,83	16,91	11,41	9,82	14,10	10,25	11,83	28,37	28,37	17,91	27,51	27,15	19,17	26,03	27,70	35,91	12,36	23,95
	4,68	10,14	7,43	6,64	9,09	6,64	5,46	9,43	7,07	5,77	9,09	7,50	4,49	17,71	12,53	4,19	13,78	11,12	12,13	15,16	13,37	19,51	12,09	12,94
L-Arginine	4,80	8,14	6,48	7,00	7,08	6,84	5,50	17,45	8,77	6,00	7,08	9,60	5,00	12,59	12,47	5,00	9,93	11,94	13,00	15,60	13,18	20,00	11,98	12,50
	4,74	11,29	9,01	6,82	8,38	8,91	5,48	11,37	10,60	5,88	8,38	9,13	4,74	14,03	11,83	4,59	11,53	11,13	12,56	13,18	13,29	19,75	11,45	12,24
	0,91	2,63	2,76	1,10	3,38	3,38	3,45	15,93	9,93	3,85	3,38	11,41	1,46	3,71	3,40	1,77	4,57	4,30	12,54	17,52	14,43	23,03	16,43	17,21
L-Glutamine	1,00	2,67	2,67	1,40	3,02	3,12	3,70	25,62	11,95	4,20	3,02	14,57	1,50	2,98	3,36	2,00	4,48	4,08	13,50	19,38	15,33	25,00	16,97	16,65
	0,95	2,64	2,65	1,25	3,14	3,29	3,58	15,15	13,02	4,03	3,14	13,18	1,48	3,70	3,28	1,89	4,72	4,88	13,02	16,46	14,48	24,01	16,28	16,63

L-Histidine	1,56	3,70	2,02	1,66	4,25	2,86	0,65	3,02	1,87	0,82	4,25	2,99	0,56	5,40	2,71	0,70	5,71	3,38	3,48	3,21	2,69	3,98	3,46	3,98
	1,70	2,38	1,62	1,90	2,92	2,49	0,75	4,23	2,74	1,00	2,92	4,20	0,75	3,08	2,81	0,80	3,32	3,98	4,00	3,79	2,95	5,00	3,58	3,78
	1,63	4,09	2,76	1,78	3,72	4,13	0,70	3,55	3,28	0,91	3,72	3,80	0,65	3,92	2,46	0,75	4,37	3,45	3,74	3,28	2,85	4,49	3,31	3,50
L-Leucine	3,55	15,51	13,93	3,27	8,45	8,81	7,22	17,46	16,11	7,16	8,45	10,33	6,60	26,15	25,87	4,32	16,64	19,59	12,94	32,44	29,01	15,81	23,30	21,34
	3,65	15,41	12,87	3,50	8,15	9,44	7,50	19,53	15,30	7,40	8,15	10,21	7,00	24,90	25,45	5,00	15,98	18,55	13,50	31,76	26,89	17,00	21,18	15,98
	3,60	15,00	14,86	3,38	8,15	9,54	7,36	17,67	15,90	7,28	8,15	10,08	6,80	26,30	25,70	4,66	17,84	18,21	13,22	30,28	25,80	16,41	21,55	20,81
L-Isoleucine	0,26	2,02	0,98	0,43	1,44	0,93	0,27	1,68	1,22	0,26	1,44	1,07	0,21	3,02	16,22	0,10	2,50	1,44	1,33	2,25	1,91	2,31	1,63	1,88
	0,35	1,30	0,83	0,60	0,94	0,83	0,40	3,85	1,52	0,60	0,94	1,53	0,30	1,95	1,87	0,30	1,39	1,76	1,50	2,58	1,88	3,00	1,78	1,39
	0,31	2,12	1,43	0,52	0,94	1,43	0,33	1,88	1,71	0,43	0,94	1,34	0,26	2,47	1,61	0,20	1,93	1,43	1,41	2,07	1,82	2,65	1,69	1,66
L-Lysine	0,67	2,15	1,71	1,07	2,03	1,74	0,55	2,40	1,58	0,92	2,03	1,81	0,53	3,38	1,92	0,54	3,90	1,78	1,86	2,85	2,66	3,06	2,67	2,09
	0,75	1,60	1,43	1,40	1,73	1,68	0,65	3,62	1,62	1,20	1,73	1,60	0,75	2,72	1,95	0,80	3,55	2,11	2,00	2,68	2,52	3,50	2,74	1,63
	0,71	2,43	2,03	1,23	1,91	1,99	0,60	2,49	2,04	1,06	1,91	1,92	0,64	3,34	1,75	0,67	3,22	2,00	1,93	2,33	2,44	3,28	2,61	1,95
L-Asparagine	0,77	0,00	0,00	1,47	0,00	0,00	0,37	3,09	4,89	0,41	0,00	3,77	0,47	0,00	0,00	0,50	0,00	0,00	2,74	1,34	8,71	5,70	1,92	6,91
	0,90	0,00	0,00	1,80	0,00	0,00	0,50	3,23	4,52	0,60	0,00	3,30	0,60	0,00	0,00	0,70	0,00	0,00	3,00	3,39	8,02	6,00	1,29	6,26
	0,84	0,00	0,00	1,64	0,00	0,00	0,43	2,18	4,00	0,51	0,00	3,72	0,54	0,00	0,00	0,60	0,00	0,00	2,87	2,75	8,14	5,85	2,50	6,50

L-Methionine	0,12	0,72	0,45	0,14	0,57	0,45	0,11	0,63	0,52	0,12	0,57	0,47	0,16	1,18	0,75	0,12	1,05	0,67	0,65	0,94	0,77	1,14	0,78	0,76
	0,20	0,52	0,40	0,30	0,41	0,43	0,20	0,78	0,61	0,30	0,41	0,62	0,25	0,85	0,76	0,30	0,64	0,81	0,70	0,95	0,78	1,40	0,81	0,74
	0,16	0,76	0,55	0,22	0,57	0,64	0,16	0,76	0,65	0,21	0,57	0,59	0,21	0,96	0,71	0,21	0,76	0,65	0,67	0,93	0,73	1,27	0,78	0,73
L-Phenylalanine	0,07	0,36	0,23	0,23	0,29	0,23	1,46	5,22	4,65	1,41	0,29	2,99	0,06	0,60	0,38	0,04	0,53	0,34	4,46	9,40	8,57	3,97	5,82	6,12
	0,15	0,26	0,20	0,40	0,21	0,22	1,60	6,45	5,24	1,60	0,21	3,10	0,10	0,43	0,39	0,07	0,32	0,41	5,00	9,04	8,32	5,00	6,07	6,11
	0,11	0,38	0,28	0,32	0,29	0,32	1,53	5,29	5,20	1,51	0,29	2,95	0,08	0,49	0,36	0,06	0,39	0,33	4,73	9,38	8,90	4,49	5,82	6,01
L-Threonine	0,39	2,23	1,20	0,74	2,21	1,49	0,22	1,38	1,31	0,22	2,21	1,63	0,25	3,76	1,64	0,14	3,54	1,88	1,85	2,04	1,64	3,64	2,08	2,30
	0,50	1,47	0,88	0,90	1,61	1,37	0,30	4,82	1,83	0,40	1,61	2,40	0,40	2,01	1,67	0,30	1,94	2,29	2,00	2,47	1,81	4,00	2,25	2,29
	0,45	2,56	1,61	0,82	2,08	2,48	0,26	2,22	2,03	0,31	2,08	2,40	0,33	2,73	1,43	0,22	2,84	1,74	1,93	1,96	1,74	3,82	2,08	2,07
L-Tryptophan	0,29	1,34	1,26	0,69	0,83	0,87	0,49	1,55	1,41	0,51	0,83	1,01	0,49	2,23	2,11	0,34	1,52	1,70	0,10	2,68	2,51	1,22	1,85	1,87
	0,40	1,37	1,19	0,90	0,82	0,93	0,65	1,63	1,30	0,30	0,82	0,99	0,50	2,12	2,12	0,50	1,44	1,69	0,30	2,22	2,37	1,60	1,79	1,78
	0,35	1,35	1,32	0,79	0,81	0,87	0,57	1,63	1,42	0,40	0,81	0,96	0,50	2,25	2,11	0,42	1,56	1,62	0,20	2,22	2,22	1,41	1,90	1,91
L-tyrosine	0,94	7,08	4,71	1,78	5,65	4,58	0,71	2,21	1,88	0,59	5,65	1,65	1,48	11,68	8,00	1,07	9,26	7,11	1,58	3,33	2,94	2,62	2,67	2,71
	1,10	5,60	4,20	2,00	4,46	4,63	0,80	2,96	2,11	0,80	4,46	2,09	1,75	8,78	8,14	1,60	6,60	7,83	2,00	3,37	2,78	3,00	2,66	2,78
	1,02	7,96	5,89	1,89	5,30	5,78	0,76	2,40	2,30	0,69	5,30	1,79	1,62	10,15	7,79	1,34	8,10	6,92	1,79	3,10	2,81	2,81	2,57	2,61

L-Valine	58,8	175,2	170,3	52,6	122,1	139,0	317,9	656,7	615,4	345,9	122,1	494,2	93,1	278,7	302,7	75,0	237,0	295,7	606,1	1160,8	1015,7	752,5	1082,5	959,1
	60,0	190,8	168,7	55,0	134,8	163,4	320,0	585,7	554,8	350,0	134,8	449,6	100,0	295,4	294,3	90,0	256,2	285,0	650,0	1079,6	938,5	800,0	992,6	923,1
	59,4	175,1	176,1	53,8	120,8	136,9	318,9	676,7	596,1	347,9	120,8	432,1	96,6	300,9	300,4	82,5	270,6	281,6	628,1	1083,2	921,0	776,3	983,7	955,0

Table S2. b: Amino acid amount (µg) detected in the apical (AP) and basolateral (BL) compartment of ThinCert inserts after **24 hours** of exposure to bio-available fraction (BAF) in the different culturing conditions. RTdiMI (RTDI) and RTpiMI (RTPI) cell lines were exposed to 25% or 50% BAF diluted in L15/ex medium supplemented with 4% BSA or without any supplementation (no BSA). The same conditions were also used on control samples without cells (NC).

BSA	BSA	BSA	BSA	BSA	BSA	BSA	No BSA	No BSA	No BSA	No BSA	No BSA	No BSA	BSA	BSA	BSA	BSA	BSA	BSA	No BSA	No BSA	No BSA	No BSA	No BSA	No BSA
**%BAF**	**25**	**25**	**25**	**25**	**25**	**25**	**25**	**25**	**25**	**25**	**25**	**25**	**50**	**50**	**50**	**50**	**50**	**50**	**50**	**50**	**50**	**50**	**50**	**50**
**Cells**	**NC**	**RTDI**	**RTPI**	**NC**	**RTDI**	**RTPI**	**NC**	**RTDI**	**RTPI**	**NC**	**RTDI**	**RTPI**	**NC**	**RTDI**	**RTPI**	**NC**	**RTDI**	**RTPI**	**NC**	**RTDI**	**RTPI**	**NC**	**RTDI**	**RTPI**
**Compartment**	**AP**	**AP**	**AP**	**BL**	**BL**	**BL**	**AP**	**AP**	**AP**	**BL**	**BL**	**BL**	**AP**	**AP**	**AP**	**BL**	**BL**	**BL**	**AP**	**AP**	**AP**	**BL**	**BL**	**BL**
L-Alanine	32,35	48,75	42,20	21,84	30,43	38,32	21,44	31,49	24,02	21,35	29,16	38,32	27,99	60,69	53,02	44,77	91,47	49,86	35,46	35,54	34,10	51,25	32,35	40,69
	35,00	48,72	49,31	23,00	25,79	39,31	22,50	32,44	30,98	23,00	23,72	31,06	30,00	63,07	58,52	46,00	79,18	47,11	36,00	39,23	33,90	53,00	33,50	40,67
	33,68	50,72	52,98	22,42	31,06	28,23	21,97	39,29	32,36	22,18	29,60	26,85	28,99	65,82	55,00	45,39	94,23	42,36	35,73	56,23	34,84	52,13	45,00	37,50
L-Arginine	20,05	21,17	21,20	20,02	20,31	16,09	13,48	18,16	16,63	16,99	16,24	35,95	18,06	24,78	22,61	24,30	45,24	21,20	18,10	28,79	27,65	24,87	24,39	24,75
	22,50	22,50	21,41	22,00	20,45	15,83	15,00	17,35	17,81	18,00	17,30	15,35	20,00	27,73	21,78	30,00	43,89	20,21	19,00	26,92	25,23	26,20	31,73	26,52
	21,27	24,33	18,36	21,01	17,31	16,27	14,24	20,10	18,88	17,50	17,73	16,62	19,03	26,73	21,66	27,15	40,65	25,32	18,55	35,99	29,92	25,53	28,40	28,20
L-Glutamine	6,56	8,11	8,78	8,54	7,06	7,61	18,98	11,94	11,43	18,72	12,89	10,94	2,89	9,24	6,77	8,65	16,75	7,83	23,78	53,86	18,05	37,46	42,05	18,01
	7,50	7,11	6,68	9,00	7,03	7,12	19,50	14,90	9,97	20,00	14,84	12,19	3,25	8,33	8,40	9,00	15,40	7,74	25,00	67,21	17,36	39,60	58,47	19,69
	7,03	9,09	6,56	8,77	7,68	6,09	19,24	13,86	12,16	19,36	13,86	12,20	3,07	8,32	6,62	8,82	17,85	7,70	24,39	72,98	18,01	38,53	53,60	20,00
L-Histidine	3,95	5,65	5,39	4,02	5,45	4,49	3,37	3,92	4,84	4,40	3,70	7,63	3,79	5,45	5,01	4,35	11,21	4,72	2,96	7,67	5,65	4,78	6,33	5,01
	4,50	5,77	4,90	5,00	5,56	4,49	4,00	4,68	4,21	5,00	4,54	3,69	4,00	5,90	4,53	5,00	12,10	4,90	3,25	7,36	5,23	5,00	8,29	6,34
	4,23	5,79	4,65	4,51	5,10	4,63	3,68	5,44	4,84	4,70	7,57	4,44	3,90	4,83	4,88	4,68	11,64	4,16	3,10	7,84	6,91	4,89	7,00	5,50

L-Leucine	21,59	22,65	18,01	20,43	17,79	13,63	4,53	4,52	4,24	4,28	3,59	3,84	23,64	28,30	27,33	26,19	54,26	22,33	3,35	16,84	6,25	4,58	13,82	4,56
	22,50	21,99	17,98	22,00	17,84	13,01	5,00	4,98	3,76	5,00	3,98	3,55	25,00	30,38	29,87	30,00	48,58	21,20	3,50	15,77	5,33	5,40	19,05	5,20
	22,04	22,73	18,55	21,21	18,55	13,03	4,77	4,88	4,08	4,64	4,03	3,83	24,32	30,05	25,97	28,10	55,57	20,74	3,42	23,47	5,74	4,99	18,40	4,80
L-Isoleucine	2,98	3,21	2,87	2,69	3,24	2,38	5,74	6,33	6,10	5,28	5,74	5,64	2,53	4,29	4,08	3,50	7,96	3,10	7,31	28,18	8,55	10,48	22,45	7,60
	3,50	3,73	2,98	2,80	3,25	2,39	6,50	6,72	6,22	6,00	5,88	5,60	3,00	4,72	4,40	4,00	7,33	2,97	8,00	26,68	8,45	12,00	31,11	7,30
	3,24	3,68	2,75	2,74	3,03	2,45	6,12	6,61	6,14	5,64	5,41	5,59	2,76	4,34	4,06	3,75	8,09	3,78	7,65	37,81	8,66	11,24	27,50	7,10
L-Lysine	6,73	7,12	5,64	6,89	6,70	4,75	2,81	2,03	1,84	2,47	2,28	2,93	5,31	8,53	5,84	8,74	16,60	5,81	2,04	7,28	2,80	3,41	2,22	2,62
	7,00	7,33	5,50	8,00	6,96	4,58	3,00	2,60	1,79	3,00	2,67	2,43	5,50	8,82	5,81	10,00	15,79	5,64	2,50	7,44	2,77	3,70	3,54	3,00
	6,87	7,20	5,07	7,44	6,26	4,22	2,90	2,71	1,81	2,73	2,70	2,37	5,40	8,06	7,33	9,37	16,00	6,00	2,27	11,24	3,28	3,55	2,60	2,80
L-Asparagine	43,18	38,99	35,60	40,49	31,36	26,02	1,38	1,35	1,92	1,24	1,42	2,13	54,67	45,81	40,76	48,98	92,29	37,45	2,18	5,61	2,15	2,27	5,24	1,68
	45,00	34,61	32,86	43,00	30,70	28,39	1,50	1,71	1,33	1,50	1,74	1,32	56,00	46,70	39,65	55,00	87,77	36,57	2,50	5,24	1,82	3,00	4,82	2,25
	44,09	38,59	31,18	41,75	33,14	27,86	1,44	2,14	1,75	1,37	2,08	1,67	55,34	47,37	38,35	51,99	93,61	34,30	2,34	5,18	2,39	2,63	4,50	1,90
L-Methionine	2,19	2,42	1,98	1,88	2,20	1,71	1,82	1,68	1,66	1,53	1,42	1,72	1,61	3,11	2,45	2,57	6,28	2,34	1,19	8,03	2,65	1,85	5,07	1,86
	2,50	2,60	2,19	2,00	2,00	1,79	2,00	1,84	1,51	2,00	1,62	1,56	2,00	3,44	2,62	3,00	6,61	2,39	1,50	7,33	2,42	2,30	7,64	2,01
	2,34	2,26	2,15	1,94	2,04	1,68	1,91	1,98	1,71	1,76	1,63	1,66	1,80	3,41	2,54	2,79	6,38	2,40	1,34	12,19	2,48	2,07	6,60	1,70
L-Phenylalanine	22,56	24,71	19,58	21,38	19,71	15,60	19,83	23,75	14,30	21,79	20,56	18,28	20,19	34,44	34,76	29,34	62,39	25,92	25,32	24,85	36,30	32,72	20,25	29,50
	25,00	24,16	19,00	23,00	19,00	15,30	21,00	23,96	22,70	24,00	19,77	19,26	21,50	33,66	31,94	34,00	61,45	22,90	26,00	23,82	35,94	34,20	33,06	26,93
	23,78	25,20	20,81	22,19	18,94	14,37	20,42	23,45	23,14	22,89	18,85	19,26	20,84	37,62	31,10	31,67	63,50	20,97	25,66	36,74	35,07	33,46	26,20	25,40
L-Threonine	2,93	4,78	3,53	2,85	4,38	3,33	1,53	1,39	2,24	1,37	1,38	2,25	2,68	4,82	4,13	3,59	9,05	3,29	1,38	5,13	2,30	2,04	4,25	1,85
	3,00	4,51	3,85	3,00	4,27	3,20	1,75	1,81	1,54	1,50	1,80	1,38	3,00	5,41	3,84	5,00	9,53	3,31	1,50	4,62	2,15	3,10	5,22	2,21
	2,97	4,36	3,53	2,93	3,53	3,07	1,64	2,31	1,87	1,43	1,79	1,86	2,84	4,43	3,68	4,30	8,96	2,18	1,44	6,49	2,80	2,57	4,20	1,50
L-Tryptophan	6,35	6,53	5,44	5,88	5,48	4,64	2,91	3,09	3,19	2,71	2,73	2,65	5,51	8,87	8,32	7,71	16,33	6,40	2,61	5,69	4,70	3,82	4,80	3,89
	12,00	6,49	5,64	6,00	5,46	4,57	3,00	3,24	3,21	3,00	2,68	2,78	6,50	8,81	7,65	9,00	16,07	6,23	3,00	5,50	4,82	4,00	7,56	3,75
	9,17	6,68	5,59	5,94	5,83	4,38	2,95	3,31	3,16	2,85	2,60	2,83	6,00	8,93	7,34	8,35	16,50	5,25	2,80	8,34	4,42	3,91	5,50	3,50
L-tyrosine	19,55	23,58	19,17	17,01	19,65	16,77	7,74	9,12	8,84	7,15	8,13	8,87	14,94	28,24	26,38	23,59	57,78	21,99	11,14	14,56	10,60	17,66	13,03	10,45
	21,00	23,23	20,06	19,00	20,41	16,73	8,00	9,66	9,40	8,00	8,21	8,06	17,00	28,96	24,75	25,00	56,52	21,30	12,00	14,50	11,02	19,20	22,94	10,08

	20,27	22,40	19,67	18,01	20,08	16,79	7,87	9,70	9,25	7,58	8,91	7,98	15,97	30,11	24,56	24,29	55,13	23,29	11,57	22,83	11,56	18,43	15,40	9,80
L-Valine	884,4	951,7	892,6	822,1	836,3	761,7	189,0	300,5	313,3	190,4	295,7	328,1	672,4	1285,9	1134,5	1122,5	2466,7	965,0	289,2	856,3	420,1	386,5	788,9	402,1
	900,0	886,7	870,7	850,0	737,5	772,2	190,0	314,7	322,7	200,0	293,1	298,4	700,0	1300,9	1163,2	1200,0	2497,2	402,2	295,0	826,9	427,4	400,3	1177,2	402,2
	892,2	982,9	922,5	836,1	883,7	733,6	189,5	309,3	304,8	195,2	276,3	289,2	686,2	1297,5	1170,1	1161,2	2407,3	503,3	292,1	1314,2	415,2	393,4	987,4	390,0

Table S2. c: Amino acid amount (μg) detected in the bio-available fraction (BAF) solution used for all the experiments and added to the system at time 0. BAF was diluted 25% or 50% in L15/ex medium supplemented with 4% BSA or without any supplementation and amino acid amount was determined.

	ALA	ARG	GLN	HIS	LEU	ILE	LYS	ASN	MET	PHE	THR	TRP	TYR	VAL
**25% BAF IN L15/EX + BSA**	29,24	16,03	16,21	1,82	1,98	35,61	3,08	1,97	0,85	11,16	1,38	3,40	8,35	455,04
**50% BAF IN L15/EX + BSA**	62,06	24,00	29,02	3,14	4,43	77,33	4,80	3,69	2,28	20,29	3,16	6,28	17,46	751,10
**25% BAF IN L15/EX**	32,24	15,58	16,16	2,30	1,94	37,42	2,72	1,91	0,83	10,62	1,44	3,25	3,27	1079,33
**50% BAF IN L15/EX**	66,85	23,00	30,88	4,25	4,18	60,39	4,63	3,53	2,38	20,04	3,13	6,05	5,74	2194,36
